# RPLP0 identified as a ferroptosis regulator in HCC through pan-cancer analysis

**DOI:** 10.3389/fonc.2026.1891222

**Published:** 2026-08-11

**Authors:** Zuli Wang, Shuai Chen, Bokang Yan, Xintong Peng, Ling Chen, Min Su, Haiyang Li

**Affiliations:** 1 Department of Hepatobiliary Surgery, Key Laboratory of Hepatobiliary and Pancreatic Diseases Treatment and Bioinformatics Research, The Affiliated Hospital of Guizhou Medical University, Guizhou Medical University, Guiyang, Guizhou, China; 2 Department of Histology and Embryology, Center for Tissue Engineering and Stem Cell Research, School of Basic Medical Science, Guiyang, Guizhou, China; 3 Hangzhou Innovation Institute, Beihang University, Hangzhou, Zhejiang, China; 4 Department of Pahtology, Affiliated Zhuzhou Hospital of Xiangya School of Medicine, Central South University, Zhuzhou, Hunan, China; 5 National Health Commission Key Laboratory of Carcinogenesis (Central South University), Key Laboratory of Carcinogenesis and Cancer Invasion (Ministry of Education), Cancer Research Institute and School of Basic Medicine, Central South University, Changsha, Hunan, China

**Keywords:** ferroptosis, GPX4, HCC, pan-cancer, RPLP0

## Abstract

**Background:**

RPLP0, a ribosomal protein critical for protein biosynthesis, has emerged as a multifaceted oncoprotein through its regulation of programmed cell death (PCD) pathways. Despite its established roles in tumorigenesis, its pan-cancer relevance and ferroptosis regulatory function remains unexplored.

**Methods:**

Bioinformatics analyses utilized TCGA, GTEx, GEPIA, UALCAN, HPA, cBioPortal, TISIDB, FerrDb, BioGRID, HitPredict, and R Studio. Experimental validation in hepatocellular carcinoma (HCC) included RT-qPCR, IHC, CCK8, transwell, and colony formation assays. Ferroptosis was assessed via lipid ROS, ferrous iron, and GSH detection.

**Results:**

Our comprehensive pan-cancer analysis revealed conserved RPLP0 upregulation across malignancies, positively associated with adverse clinical outcomes. Genomic profiling identified recurrent RPLP0 alterations, while tumor microenvironment analyses demonstrated significant negative correlations between RPLP0 expression and immune cell infiltration. Focusing on hepatocellular carcinoma (HCC), experimental validation confirmed that RPLP0 depletion suppressed tumor progression via ferroptosis induction through metabolic and oxidative cascades, and RPLP0 was positively correlated with key ferroptosis suppressor GPX4.

**Conclusions:**

This work advances the understanding of ribosomal protein biology by establishing RPLP0 as a pan-cancer biomarker bridging ribosome function with immune evasion, unveiling its canonical role in HCC progression, and discovering RPLP0-GPX4-mediated ferroptosis resistance axis, providing a novel therapeutic paradigm for aggressive cancers.

## Introduction

Cancer is probably the most dreaded disease, with about 20 million people diagnosed with cancer worldwide each year (1). Even with major advances in curative alternatives such as chemoradiotherapy, molecular targeted therapy and immunotherapy, the morbidity and mortality rates of cancer remain high (2–4). Thus, it is critical to identify novel biomarkers and therapeutic targets for cancers.

Ribosomal Protein Lateral Stalk Subunit P0 (RPLP0), a component of the human ribosome P complex, accelerates protein synthesis by recruiting translation factors (5, 6). Many studies prove that RPLP0 is an essential modulator of many diseases such as Alzheimer’s disease, lupus skin lesions and especially cancers (7–9). It has been reported that high expression levels of RPLP0 suppresses cell apoptosis in NSCLC, gastric cancer and cervical cancer (9–11). Additionally, RPLP0 contributes to the radioresistance of rectal cancer by interacting with NONO (12), and inhibits the autophagy of gynecologic cancer cells (13). These findings indicate that RPLP0 is a potentially valuable target for tumor diagnosis and therapy. However, a comprehensive analysis of the role of RPLP0 in pan-cancer has not been performed yet.

Ferroptosis is a novel type of regulated cell death marked by the accumulation of reactive oxygen species (ROS) (14). Studies have proven that the high susceptibility of cancer cells to ferroptosis offers great opportunities for cancer treatment (15, 16). Ferroptosis-regulating proteins, such as GPX4, p53, and SLC7A11, have been demonstrated to exhibit remarkable effects on regulating of tumor progression (17–19). RPLP2, a paralog of RPLP0 within the ribosomal P-complex, suppresses ferroptosis in Hepatocellular carcinoma (HCC) by stabilizing GPX4 (20), and positively correlated with FXN to inhibit ferroptosis in diffuse large B-cell lymphoma (DLBCL) (21). And RPLP0 knockdown has also been reported to lead to ROS accumulation in gynecological tumors (13). However, the specific function of RPLP0 in ferroptosis remains unexplored.

Therefore, this leads us to explore RPLP0’s pan-cancer oncogenic roles, particularly its carcinogenic impact on HCC, and to elucidate how RPLP0 modulates cellular redox balance to regulate ferroptosis susceptibility, potentially unveiling novel therapeutic strategies for refractory cancers.

## Materials and methods

### Gene expression analysis

The mRNA data of RPLP0 in pan-cancer and corresponding para-cancer or normal samples were downloaded from The Cancer Genome Atlas (TCGA) and Genotype-Tissue Expression (GTEx) databases. The correlations between RPLP0 expression and pathological stages were analyzed via Expression Profiling Interactive Analysis (GEPIA). The National Cancer Institute’s CPTAC dataset was applied to analyze the protein levels of RPLP0 in different cancers. And the subcellular locations of RPLP0 were presented by COMPARTMENTS and Human Protein Atlas (HPA) databases. Statistical analysis was performed by R software (v 3.6.3), and visualization was conducted via the “ggplot2” (v3.3.3) package.

### Prognostic and diagnostic value analysis

The ‘forestplot’ package was used for visualization of the forest map to present the P value, HR and 95% CI of each variable of univariate Cox regression. And the diagnostic ROC curve was performed using the “pROC” (v1.17.0.1) package to analyze the diagnostic value of RPLP0 in pan-cancer, and plotting was presented by the “ggplot2” (v3.3.3) package. The closer the area under the curve (AUC) was to 1, the higher the diagnostic accuracy.

### Genetic alteration and DNA methylation analysis

The cBioPortal was applied to search for the gene alternations of RPLP0 in TCGA PanCancer Atlas Studies. Through the modules “Cancer Type Summary”, “Mutations” and “Survival”, we explored the status of genetic alterations and mutation site and the effects of RPLP0 gene alterations on the prognosis of all cancer patients. And the promoter methylation features of RPLP0 in cancers were studied in the UALCAN database.

### Tumor mutational burden, microsatellite instability and immunogenomic analyses

Downloaded RPLP0 RNA-sequencing expression profile and corresponding clinical information from TCGA. TMB was derived from an article published by Vesteinn Thorsson et al. in 2018: the Immune Landscape of Cancer (22); MSI was derived from Russell Bonneville et al. ‘s the Landscape of Microsatellite Instability Across 39 Cancer Types published in 2017 (23). XCELL algorithm was applied to assess the correlation between RPLP0 expression and immune cell infiltration levels. The expression value of immune-checkpoint-related genes were analyzed by extracting the expression of SIGLEC15, TIGIT, CD274, HAVCR2, PDCD1, CTLA4, LAG3 and PDCD1LG2. And we also explored the relationships between RPLP0 expression and chemokines or chemokine receptors in pan-cancer via the TISIDB database.

### Cell culture, plasmids, siRNAs, and chemicals

HCC cell lines HepG2, Hep3B and Huh-7 were provided by the Cancer Research Institute of Central South University. WRL68 and JHH-2 were acquired from The American Type Culture Collection (ATCC). Cells were maintained in DMEM medium (Gibco, USA) with 10% fetal bovine serum (FBS) and cultured in an incubator at 37 °C with 5% CO_2_.

RPLP0-siRNA was provided by GenePharma, Shanghai. We transfected siRNA using Lipofectamine^®^2000 (Invitrogen) according to manufacturer’s instructions.

Ferroptosis inducer RSL-3 (S8155) and ferroptosis inhibitor Ferrostatin-1 (Fer-1)(S7243) were purchased from Selleck.

### RT-qPCR

Total RNA was isolated by TRIzol (Takara, Kusatsu, Japan) and cDNA was synthesized by PrimeScriptTM RT reagent kit containing gDNA Eraser (Takara, Kusatsu, Japan). RT-qPCR was performed using ABI 7500 and FastStart Universal SYBR Green Master. To normalize the expression levels of related genes, β-actin was employed. The sequences of RT-qPCR primers were shown in **Supplementary Table 1**.

### Immunohistochemistry

The complete procedure has been described in this manuscript before (24). HCC patient tissues were accessed at Zhuzhou Central Hospital from 05/01/2016 to 03/01/2024 with institutional ethical approval for research purposes. The primary antibodies applied for IHC in this study included RPLP0 (1:200, 11290-2-AP, Proteintech), 4-HNE (1:100, ab46545, Abcam), and GPX4 (1:100, ab125066, Abcam). Bokang Yan, a pathological technician at Zhuzhou Central Hospital’s Department of Pathology, verified the liver cancer biopsies.

### Cell proliferation, migration, and colony formation assays

Cell viability was analyzed using the CCK8 kit (TP1197, Topscience) according to the manufacturer’s instructions. 3×10^4^ cells were implanted in a chamber for migration assays. After 24 hours, the cells on the upper surface of the chamber were removed, then cells were fixed and stained with 0.5% crystal violet. In the colony formation experiment, cells were seeded into the six-well plate at a density of 1,000 cells per well. After about two weeks, the cells were fixed with methanol and stained with 0.5% crystal violet.

### Co-immunoprecipitation assay

Co-immunoprecipitation (Co-IP) assay was performed in HepG2 cells to examine the physical interaction between RPLP0 and GPX4. Briefly, cells were rinsed with pre-cooled PBS and lysed in non-denaturing IP lysis buffer supplemented with protease inhibitor cocktail on ice for 30 min. The lysate was centrifuged at 12000 × g for 15 min at 4 °C, and the supernatant was harvested as total protein lysate. An aliquot of supernatant was retained as the Input control.

The remaining lysate was split equally into two tubes: one incubated with GPX4 primary antibody, and the other incubated with species-matched normal IgG as negative control. The mixtures were rotated overnight at 4 °C. After antibody binding, magnetic Protein A/G beads were added to each tube, followed by 4 h incubation at 4 °C with gentle rotation to capture antibody-protein complexes.

A magnetic rack was used to separate the magnetic beads from the liquid phase. The beads were washed five times with pre-chilled IP lysis buffer to eliminate non-specifically bound proteins. Captured protein complexes were denatured and eluted by resuspending beads in 1× SDS loading buffer and heating at 95 °C for 10 min. Finally, Input, IgG control and eluted IP samples were subjected to western blot to detect RPLP0 and GPX4 protein bands.

### Western blotting

Total protein from HepG2 and Hep3B cells was extracted using RIPA lysis buffer supplemented with protease inhibitor cocktail. Protein concentration was quantified via BCA assay, and equal amounts of protein samples were separated by 12% SDS-PAGE gel electrophoresis. Electrophoresis was run at a constant voltage of 80 V until protein markers reached the appropriate separation position. Afterwards, proteins were transferred onto 0.22 μm PVDF membranes under a constant current of 400 mA for 60 min. After transfer, membranes were blocked with 5% non-fat milk in TBST buffer at room temperature for 1 h, followed by incubation with primary antibodies against RPLP0, GPX4 and β-Actin overnight at 4 °C. After three washes with TBST, membranes were incubated with corresponding HRP-conjugated secondary antibodies for 1 h at room temperature. Protein bands were visualized using enhanced chemiluminescence (ECL) reagent.

### Measurement of lipid ROS and total GSH and iron assay

For the lipid ROS assay and total GSH detection, the complete protocols had been introduced in the manuscript published before (24). Cells were treated with 10 µM C11-BODIPY (D3861, Thermo Fisher) for the lipid ROS measurement. And the total GSH detection was performed using GSH and GSSG Assay Kit (S0053, Beyotime).

The experimental method for detection of ferrous iron (Fe2+) and total iron had been described in this manuscript (25). The Iron Assay Kit (Sigma-Aldrich, USA) were applied for this experiment.

### Statistical analyses

All experiments except subcutaneous tumor formation experiments were performed at least three times. The presenting form of data was the mean ± SD or SEM. GraphPad Prism 8.0 and Microsoft Excel were conducted for statistical analysis. If not stated otherwise, the student’s t test and the analysis of variance (ANOVA) were applied for comparing two or more groups. P-value < 0.05 was considered statistically significant.

## Results

### Expression profiles of RPLP0 in pan-cancer

**Figure 1:**
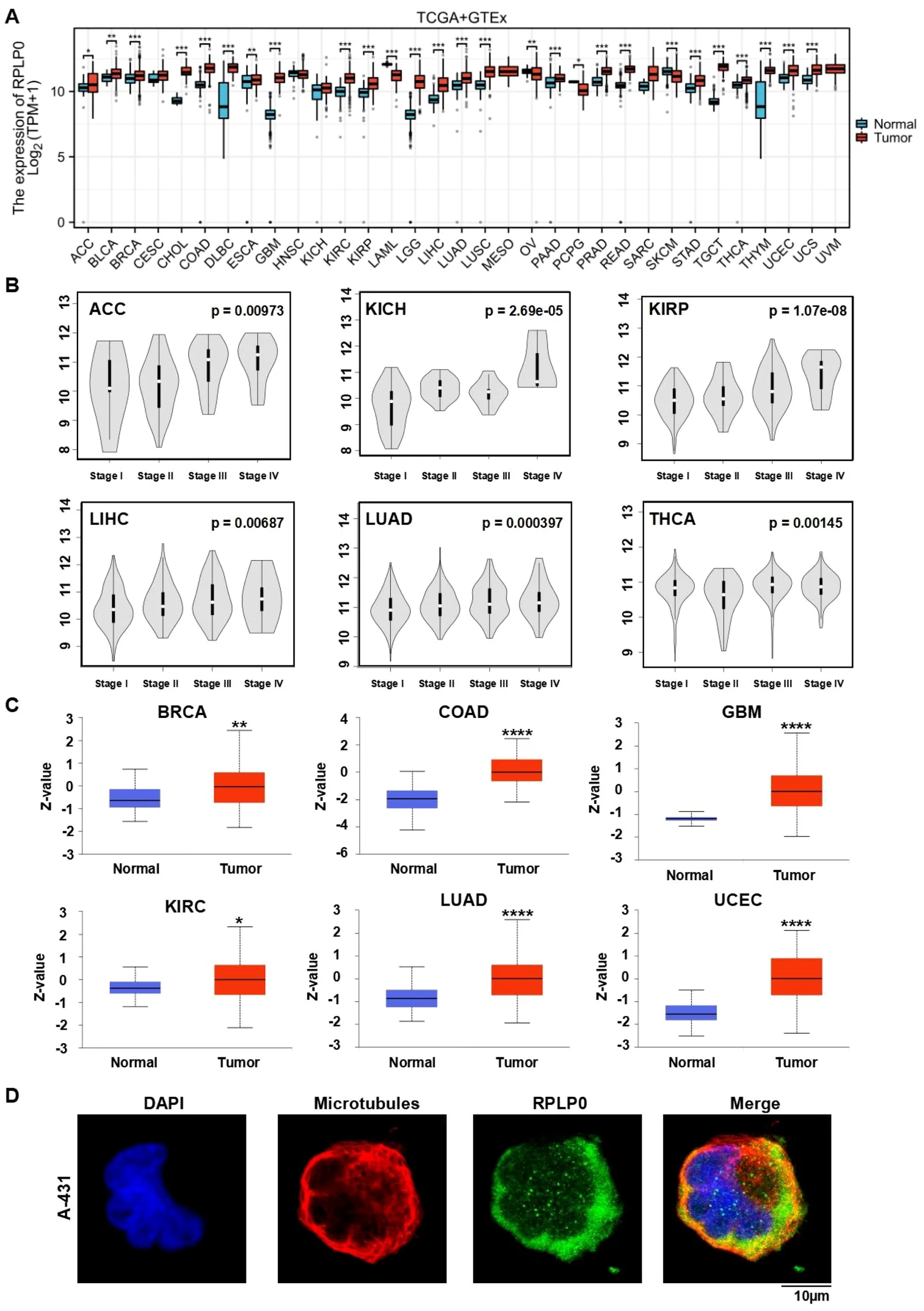
RPLP0 expression in pan-cancer. **(A)** The expression of RPLP0 in pan-cancer from TCGA and GTEx. **(B)** RPLP0 expression in different pathological stages from GEPIA. **(C)** The protein level of RPLP0 in different cancers was analyzed using CPTAC database. **(D)** Immunofluorescence staining of RPLP0 and microtubules in A-431 cells obtained from HPA database. *p < 0.05, **p < 0.01, ***p < 0.001, ****p < 0.0001.

Initially, we evaluated the mRNA expression levels of RPLP0 in pan-cancer, and the results indicated that compared with normal tissues, RPLP0 was significantly upregulated in ACC, BLCA, BRCA, CHOL, COAD, DLBC, ESCA, GBM, KIRC, KIRP, LGG, LIHC, LUAD, LUSC, PAAD, PRAD, READ, STAD, TGCT, THCA, THYM, UCEC and UCS, and low RPLP0 expression was only observed in LAML, OV, PCPG and SKCM (**Figure 1A**). Moreover, the result of GEPIA analysis showed RPLP0 mRNA expression was upregulated and positively correlated with the pathological stages in ACC, KICH, KIRP, LIHC, LUAD and THCA (**Figure 1B**). Furthermore, we found the protein level of RPLP0 was also higher in BRCA, COAD, GBM, KIRC, LUAD and UCEC by using the National Cancer Institute’s CPTAC dataset (**Figure 1C**). At last, we explored the subcellular localization of RPLP0 based on COMPARTMENTS and HPA databases. The results obtained from COMPARTMENTS exhibited that RPLP0 was mainly located in cytosol, extracellular and nucleus (**Supplementary Figure 1A**). RPLP0 mainly distributed in the cytosol and endoplasmic reticulum in A-431 (epidermoid carcinoma cell line) and U-251MG (astrocytoma cell line) cells showed by HPA (**Figure 1D**, **Supplementary Figure 1B**).

### Prognostic and diagnostic potential of RPLP0 in pan-cancer

**Figure 2:**
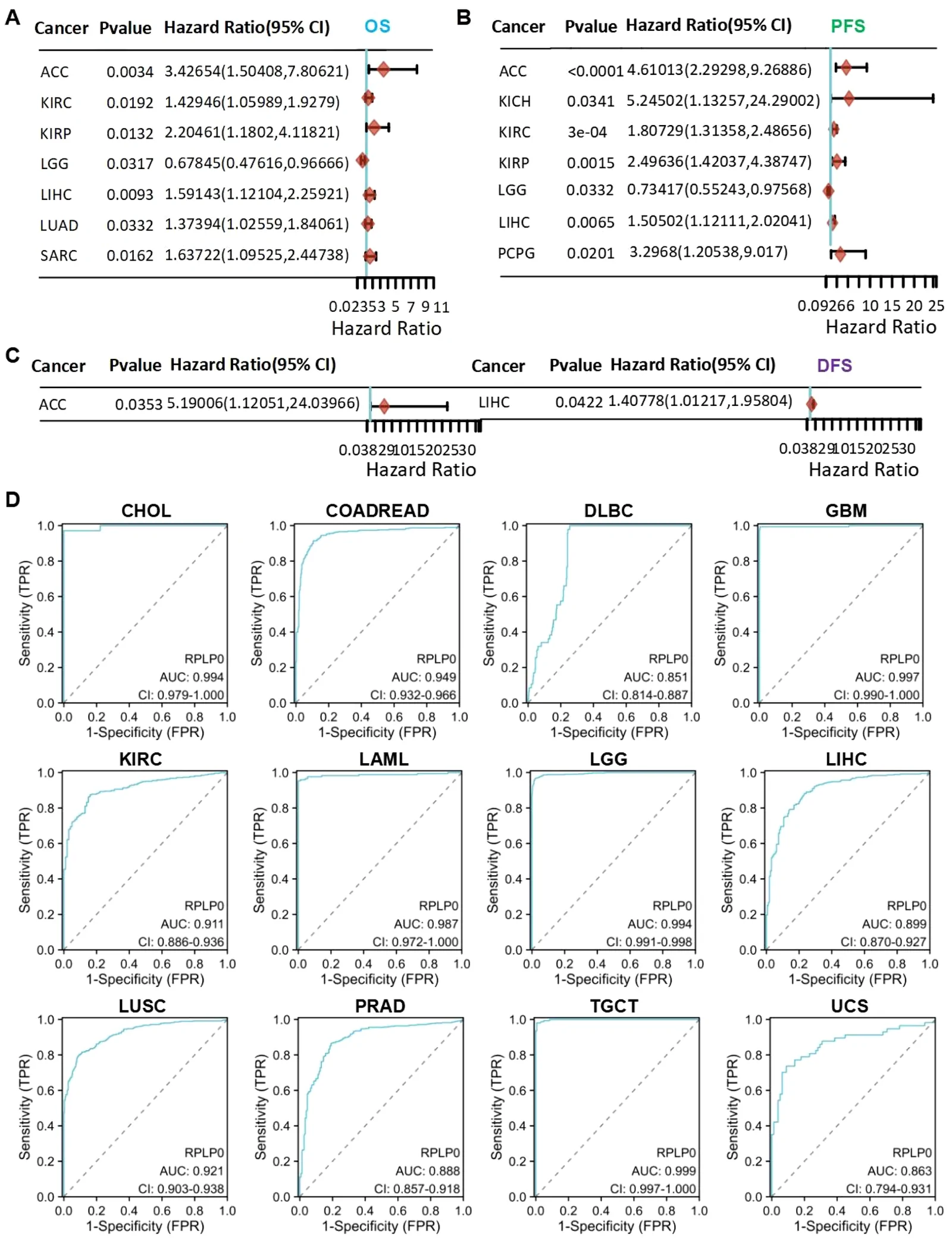
Prognostic and diagnostic value of RPLP0 in pan-cancer. **(A-C)** Forest plots exhibited the correlation between RPLP0 and OS **(A)**, PFS **(B)**, or DFS **(C)** in different cancers. **(D)** ROC curve analysis of RPLP0 diagnostic potential in pan-cancer. Data were obtained from TCGA database.

To assess the prognostic value of RPLP0 in pan-cancer, we used the univariate cox regression analysis and the forest plot to discover that patients with high level of RPLP0 had shortened overall survival (OS) in ACC, KIRC, KIRP, LIHC, LUAD and SARC (**Figure 2A**, **Supplementary Figure 2A**). For progression-free survival (PFS), patients with increased RPLP0 expression exhibited poor outcome in ACC, KICH, KIRC, KIRP, LIHC and PCPG (**Figure 2B**, **Supplementary Figure 2B**). Additionally, RPLP0 was a risk factor for disease-free survival (DFS) in ACC and LIHC (**Figure 2C**, **Supplementary Figure 2C**). To further investigate the diagnostic value of RPLP0 in pan-cancer, we conducted ROC curve analysis to demonstrate that RPLP0 showed certain diagnostic performance in 21 cancer types (AUC > 0.7), particularly in CHOL, COADREAD, DLBC, GBM, KIRC, LAML, LGG, LIHC, LUSC, PRAD, TGCT and UCS (AUC > 0.85) (**Figure 2D**, **Supplementary Figures 3A–S**). In conclusion, RPLP0 was mainly correlated to poor prognosis and showed high diagnostic accuracy in pan-cancer.

### Genetic alteration and promoter methylation of RPLP0 in pan-cancer

**Figure 3:**
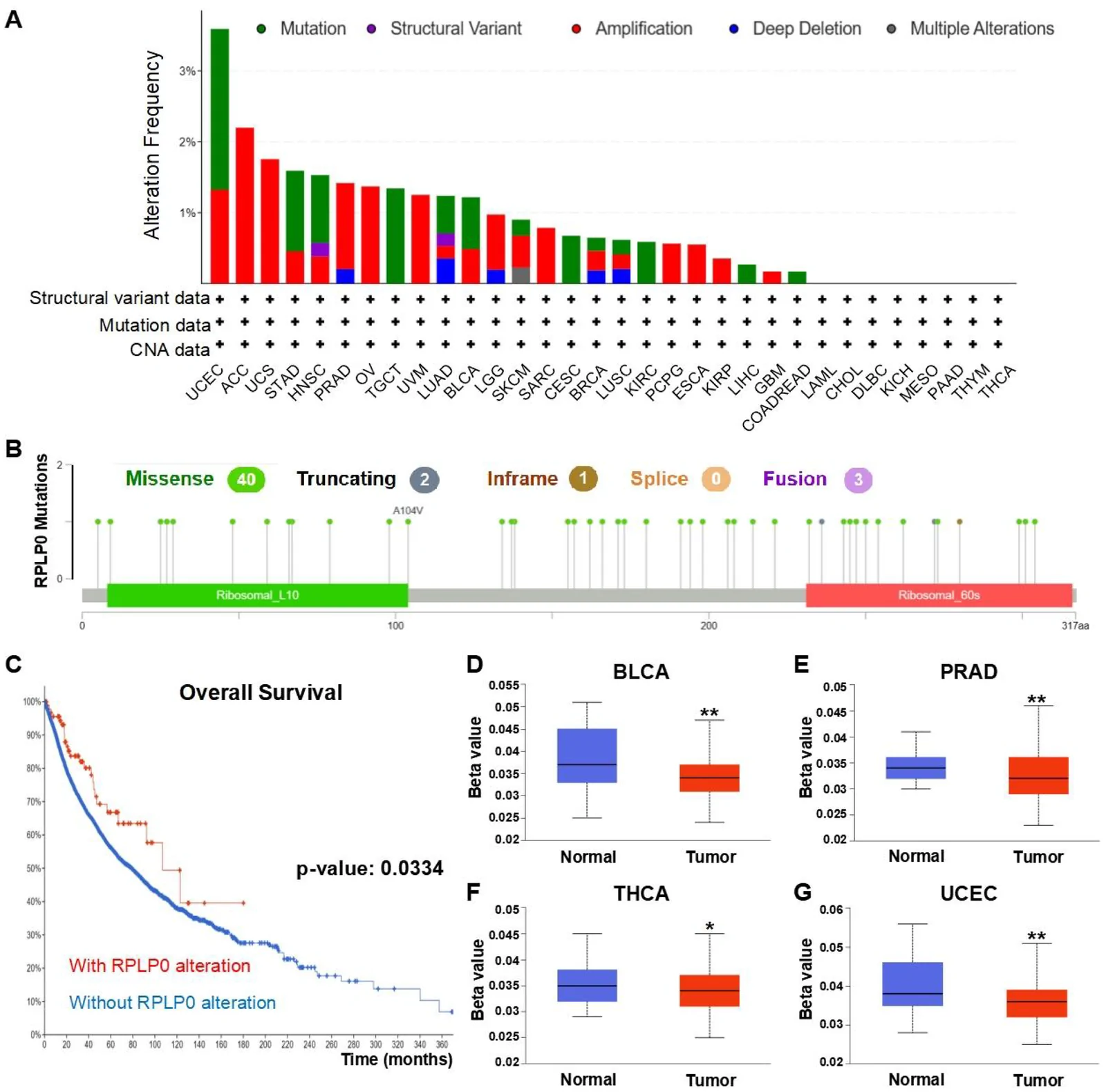
The genetic alterations and DNA methylation levels of RPLP0 in pan-cancer. **(A)** Genetic alteration frequency of RPLP0 in TCGA Pan-Cancer Atlas studies. **(B)** Mutation diagram of RPLP0 in pan-cancer. **(C)** Kaplan-Meier curve showing the association between RPLP0 mutation status and OS of cancer patients. **(D–G)** Promoter methylation levels of RPLP0 in BLCA **(D)**, PRAD **(E)**, THCA **(F)** and UCEC **(G)** analyzed via UALCAN database. *p < 0.05, **p < 0.01.

The genetic mutations of RPLP0 in various cancers were explored based on TCGA PanCancer Atlas Studies through cBioPortal online platform. RPLP0 mutations appeared most frequently in UCEC, ACC, UCS, STAD, HNSC and PRAD, and the most predominant genetic alteration types of RPLP0 were mutation, amplification and deep knockdown (**Figure 3A**). Additionally, 46 mutation sites including 40 missense mutations, 2 truncating, 1 inframe and 3 fusion were presented in **Figure 3B**. Moreover, the survival analysis results indicated that patients with RPLP0 alteration had better OS in pan-cancer (**Figure 3C**), but not disease-specific survival (DSS), PFS and DFS (**Supplementary Figures 4A–C**). Considering the significant impact of DNA methylation on transcriptional repression and cancer oncogenesis, we further analyzed the promoter methylation level of RPLP0 in pan-cancer through the UALCAN database. The analysis results displayed that the promoter methylation level of RPLP0 significantly reduced in BLCA, PRAD, THCA and UCEC (**Figures 3D–G**), and increased in COAD, KIRC, LUSC, PAAD and SARC (**Supplementary Figures 5A–E**).

### Immunogenomic analyses of RPLP0 in pan-cancer

**Figure 4:**
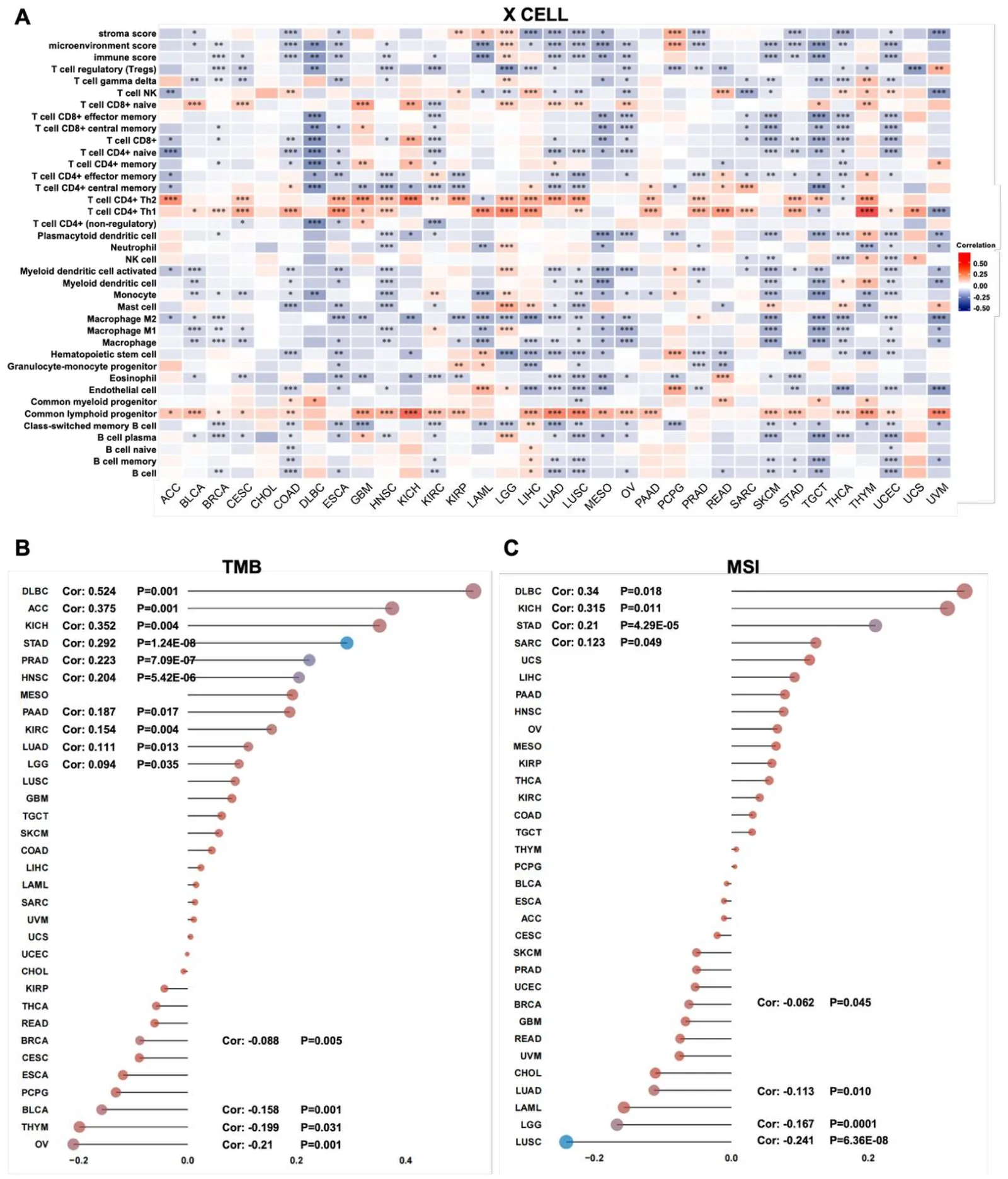
Correlation of RPLP0 with TILs, TMB, and MSI in pan-cancer. **(A)** The heatmap revealed the correlation between RPLP0 expression and immune cell infiltration in pan-cancer. **(B, C)** Lollipop plots showing correlations between RPLP0 expression and TMB **(B)** or MSI **(C)**. Data were obtained from TCGA database. *p < 0.05, **p < 0.01, ***p < 0.001.

To investigate the correlation between RPLP0 expression and immune cell infiltration or immune regulation, we drew heatmaps of RPLP0 with markers of immune cells or factors. The results showed that the microenvironment scores were significantly negatively correlated with RPLP0 expression in 18 of 33 cancers including BLCA, BRCA, COAD, DLBC, ESCA, HNSC, LAML, LIHC, LUAD, LUSC, MESO, OV, PRAD, SKCM, STAD, TGCT, THCA and UCEC, and positively associated with RPLP0 in only 2 of 33 cancers including, LGG and PCPG. Additionally, RPLP0 was negatively linked to most immune cells in pan-cancer except Naïve CD8+ T cell, Th2 CD4+ T cell, Th1 CD4+ T cell and common lymphoid progenitor (**Figure 4A**). In terms of immune checkpoint, a negative correlation between RPLP0 and immune checkpoint related genes (ICGs) could be observed in most cancer types except KIRP and LIHC (**Supplementary Figure 6A**). As for chemokines and chemokine receptors, we found that most chemokines with the exception of CCL1, CCL16, CCL24, CCL25 and CCL27 (**Supplementary Figure 6B**) and the majority of chemokine receptors except CCR3, CCR9 and XCR1 (**Supplementary Figure 6C**) exhibited certain relationships with RPLP0 in multiple cancers.

### TMB and MSI analyses of RPLP0 in pan-cancer

Both TMB and MSI are potential biomarkers for predicting the efficacy of immune checkpoint inhibitors (ICIs) (26, 27), so we further analyzed the correlation of RPLP0 with TMB or MSI. The results indicated that RPLP0 was significantly positively correlated with TMB in 10 of 33 cancer types (DLBC, ACC, KICH, STAD, PRAD, HNSC, PAAD, KIRC, LUAD and LGG), especially in DLBC (correlation coefficient (Cor) = 0.524), and negatively linked to TMB in 4 of 33 cancer types (BRCA, BLCA, THYM, OV) (**Figure 4B**). For MSI, RPLP0 was positively related to DLBC, KICH, STAD and SARC, and negatively associated with LUSC, LGG, LUAD and BRCA. Among 8 cancer types mentioned above, DLBC still had the highest correlation coefficient (Cor = 0.34) with RPLP0 (**Figure 4C**).

### RPLP0 knockdown suppresses HCC progression

**Figure 5:**
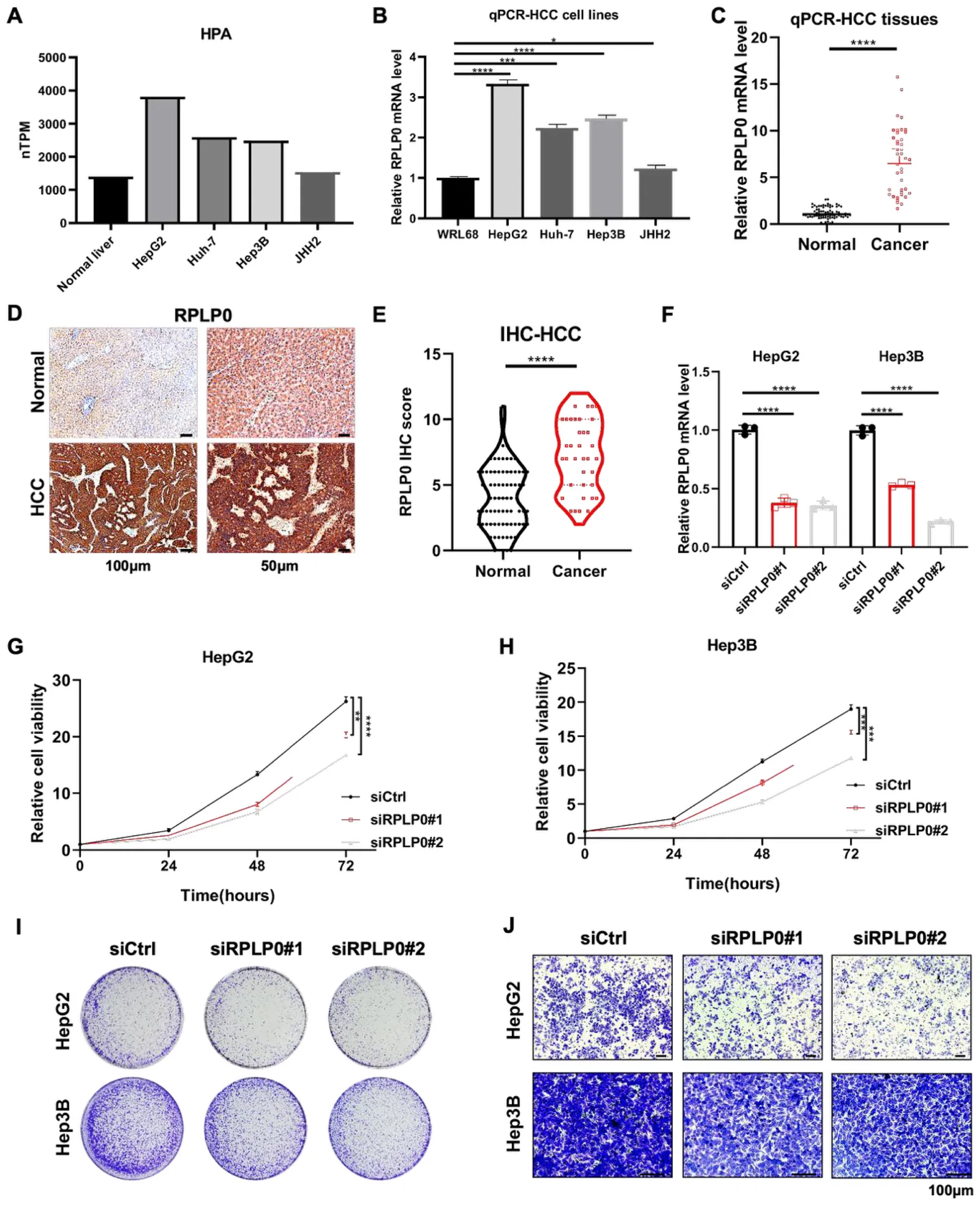
Knockdown of RPLP0 in HCC cells suppresses tumor self-renewal. **(A)** HPA database revealed RPLP0 mRNA expression level in normal liver and HCC cell lines. **(B)** RT-qPCR for verifying RPLP0 high mRNA expression level in HCC cell lines. **(C)** RT-qPCR for verifying RPLP0 mRNA expression in the Zhuzhou Central Hospital HCC cohort (n = 152), including normal liver tissues (n = 76) and HCC tissues (n = 76). **(D)** IHC analysis of RPLP0 expression in Zhuzhou Central Hospital HCC cohort. **(E)** IHC scores indicating RPLP0 levels in normal liver tissues and HCC tissues. **(F)** RT-qPCR validation of RPLP0 knockdown efficiency in HepG2/Hep3B cells. **(G, H)** The CCK8 analysis represented RPLP0 knockdown inhibited HepG2 **(G)** and Hep3B **(H)** cells proliferation. **(I)** Silence of RPLP0 suppressed clone formation capability of HCC cells showed by the colony formation assay. **(J)** Transwell migration assay of HCC cells with RPLP0 knockdown. *p < 0.05, **p < 0.01, ***p < 0.001, ****p < 0.0001.

To verify the oncogenic effects of RPLP0, we chose HCC as a typical cancer type for further study. First, we used HPA database to show that RPLP0 was upregulated in HCC cell lines by comparing with normal liver cells (**Figure 5A**), and the result of RT-qPCR further demonstrated RPLP0 exhibited higher mRNA level in HCC cell lines (**Figure 5B**) and HCC clinical tissues (**Figure 5C**). As for protein expression, IHC analysis indicated that RPLP0 was raised in HCC tissues compared to normal liver tissues (**Figures 5D, E**), Full clinicopathological data of all HCC subjects involved in RT-qPCR and IHC detection are summarized in **Supplementary Table 4**, covering age, gender, viral infection status, cirrhosis, AFP, tumor differentiation and TNM stage. This table separately sorts information of 76 RT-qPCR pairs and 12 qualified IHC pairs, and we discuss the reason for inconsistent sample quantities in the study limitations. To further investigate the functions of RPLP0 in HCC, we silenced RPLP0 in HepG2 and Hep3B cells (**Figure 5F**). The CCK8 analysis demonstrated that the knockdown of RPLP0 markedly decreased cell proliferation (**Figures 5G, H**). Moreover, colony formation assay revealed that RPLP0 silencing significantly inhibited cell survival (**Figure 5I**). Additionally, transwell assays showed that RPLP0 knockdown dramatically suppressed the migration capacity of HCC cells (**Figure 5J**). Conclusively, these results suggested that RPLP0 was upregulated in HCC tissues and RPLP0 knockdown inhibited HCC progression notably.

### Knockdown of RPLP0 promotes ferroptosis of HCC cells

**Figure 6:**
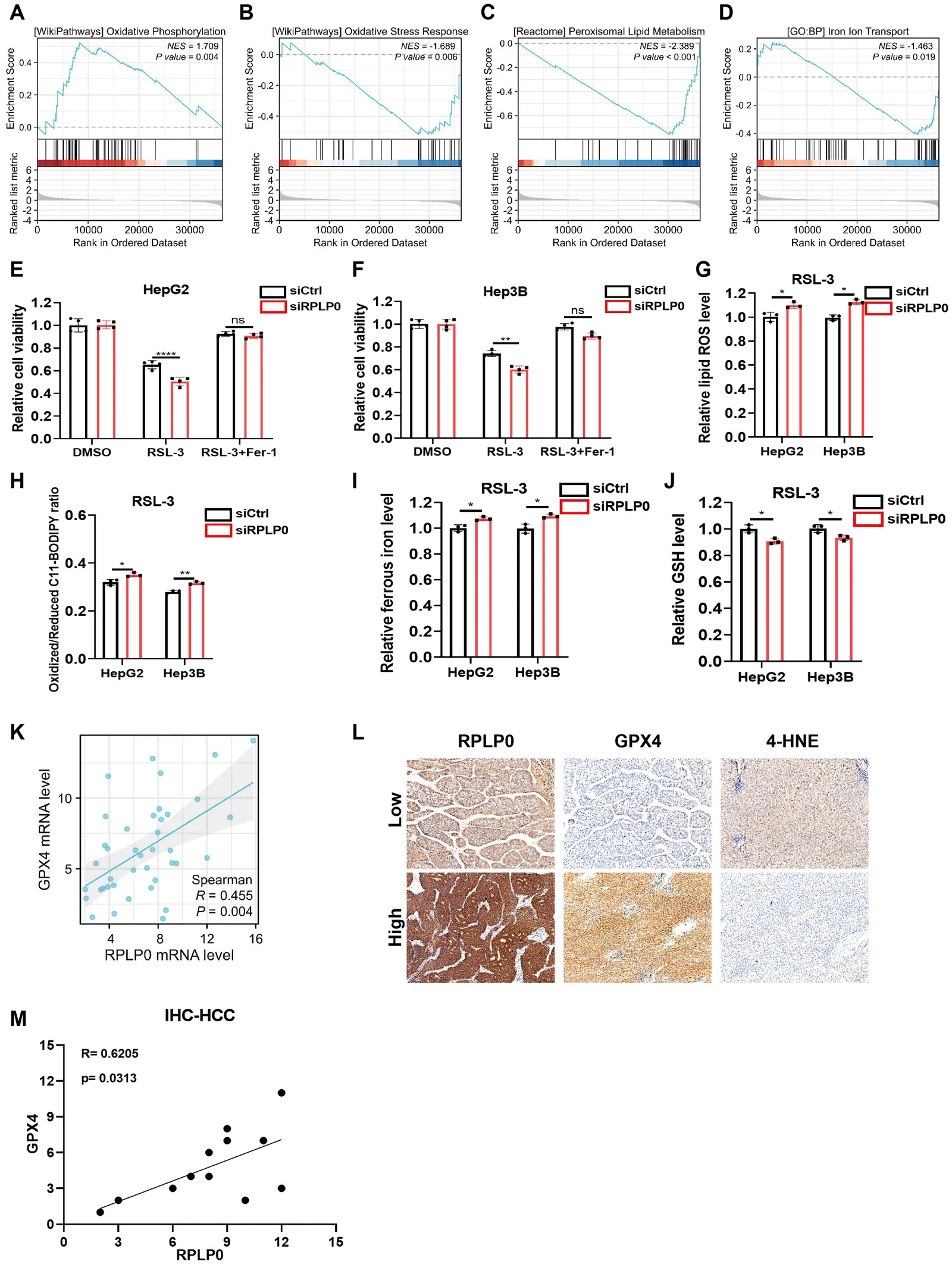
RPLP0 knockdown promotes ferroptosis of HCC cells. **(A–D)** GSEA analysis of TCGA data enriched the ferroptosis-related pathways. **(E, F)** Using CCK8 assays, the responses of HepG2 **(E)** and Hep3B **(F)** cells with RPLP0 knockdown to RSL-3 and Fer-1 were detected. **(G–J)** Levels of lipid ROS **(G)**, Oxidized/reduced C11-BODIPY ratio **(H)**, ferrous iron **(I)** and GSH **(J)** in RPLP0 knockdown HCC cells. **(K)** Spearman correlation between RPLP0 and GPX4 in 40 clinical HCC specimens. **(L)** IHC staining of RPLP0, GPX4 and 4-HNE expression in 12 clinical HCC tissues. **(M)** Correlation between RPLP0 and GPX4 protein level in 12 clinical HCC tissues. ns, nonsignificant (p > 0.05), *p < 0.05, **p < 0.01, ****p < 0.0001.

Inhibition of RPLP0 has been proved to induce ROS accumulation in gynecological tumors (13), meanwhile RPLP2, also a component of ribosomal P complex, could regulate ferroptosis of HCC and DLBCL cells (20, 22). Thus, we further explored whether RPLP0 had an impact on ferroptosis. We first conducted Gene set enrichment analysis (GSEA) to find that RPLP0 expression was significantly correlated with several ferroptosis-related pathways including “Oxidative phosphorylation”, “Oxidative stress response”, “Peroxisomal lipid metabolism” and “Iron ion transport” (**Figures 6A–D**). Then, we treated HepG2 and Hep3B cells knockdown of RPLP0 with RSL-3, and the results of morphological observations (**Supplementary Figures 7A, B**) displayed typical morphological characteristics of ferroptosis after RPLP0 silencing in HCC cells, including obvious cell shrinkage, cytoplasmic blebbing, cell rounding and loss of normal adherent morphology, which are classic cellular phenotypic hallmarks of ferroptotic cell death, and CCK8 analysis (**Figures 6E, F**) indicated that RPLP0 knockdown promoted the ferroptosis of HCC cells. Moreover, we observed that RPLP0 silencing increased the intracellular lipid ROS (**Figure 6G**) and ferrous iron (**Figure 6H**), and decreased total GSH (**Figure 6I**). Additionally, we analyzed the correlation between RPLP0 and ferroptosis regulators, and the results from FerrDb showed that RPLP0 was significantly positively associated with essential ferroptosis suppressor GPX4 (**Supplementary Figures 7C–G**). Meanwhile, the results of RT-qPCR (**Figure 6J**) further demonstrated the positive correlation between RPLP0 and GPX4, and IHC assay utilizing 12 clinical tissue pairings also proved that RPLP0 was positively linked to GPX4 and negatively related to 4-HNE, a biomarker of ferroptosis (**Figures 6K, L**). Together, these findings indicated that RPLP0 knockdown promoted ferroptosis of HCC cells.

To further evaluate RPLP0’s translational value, we conducted pharmacogenomic analyses using CellMiner, GDSC and CCLE databases. All correlation statistics for antitumor drugs are listed in **Supplementary Table 3**. RPLP0 was strongly correlated with Ribavirin and Palbociclib in CellMiner, positively associated with Daporinad and Ispinesib in GDSC, and negatively correlated with sorafenib (ρ = –0.308, FDR < 0.001) in CCLE, connecting RPLP0 to ferroptosis and immune regulation.

## Discussion

**Figure 7:**
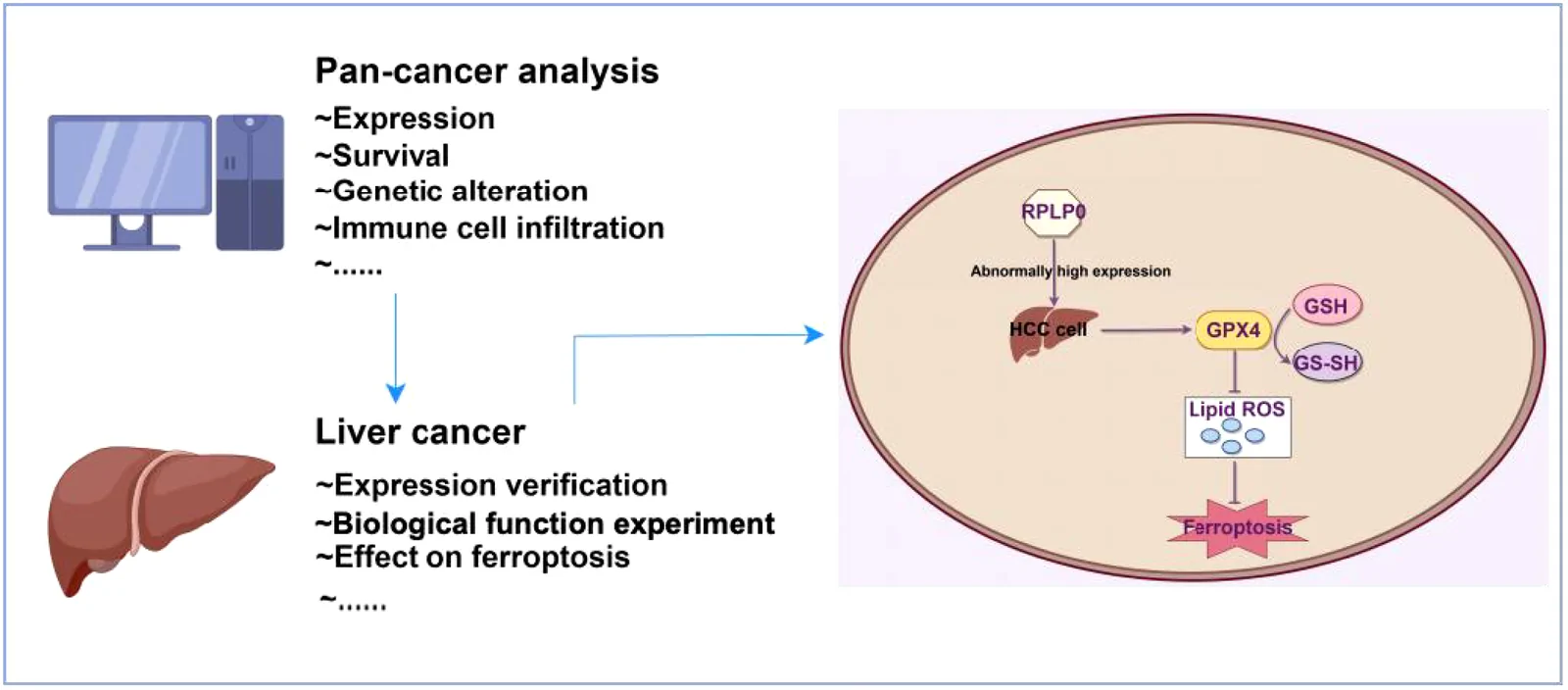
Schematic overview of the experimental workflow.

RPLP0, an important ribosome stalk protein, plays a critical role in the regulation of protein synthesis (5). Increasing studies indicate that RPLP0 could participate in tumor progression through regulating different types of programmed cell death (PCD) such as inhibiting apoptosis of cancer cells in NSCLC, gastric cancer and cervical cancer and suppressing autophagy of gynecologic cancer cells (9–11, 13). However, the regulatory effect of RPLP0 in pan-cancer remains unclear, and the concrete role and mechanism of RPLP0 in ferroptosis has not been explored. Here, we first investigated the expression level and prognostic value of RPLP0 in various cancers. Then, we revealed the gene alterations and methylation levels of RPLP0 in pan-cancer. In addition, we conducted correlation analyses between RPLP0 expression and infiltration level of different immune cells and regulators. Furthermore, the oncogenic role of RPLP0 was verified in HCC, and the specific effect of RPLP0 on ferroptosis was elucidated (**Figure 7**). Building on bioinformatic screening, we supplemented core cellular experiments including western blot, GPX4/GCLC dual rescue and magnetic bead-based Co-IP to clarify the indirect RPLP0-GPX4 regulatory cascade.

Previous studies have demonstrated that RPLP0 is mainly overexpressed and plays a tumorigenic role in cancer progression (9, 26). Consistent with their researches, our results based on several databases showed that RPLP0 expression level was relatively higher in a majority of cancers than in corresponding normal tissues. Additionally, OS, PFS and DFS analysis presented that a high RPLP0 expression was significantly correlated with poor prognosis in many cancer types, especially ACC and LIHC. Moreover, RPLP0 showed at least moderate diagnostic accuracy in most cancers (21 of 32). Since we realized the potential cancer-promoting effect of RPLP0 in pan-cancer, including LIHC, we further chose HCC to validate its oncogenic effect. And a series of *in vitro* biological function experiments proved that RPLP0 contributes to the proliferation and migration ability of HCC cells. In summary, these results suggested that RPLP0 primarily played a key role in promoting the development of tumors, which could be an important diagnostic and therapeutic target for cancers. We also supplemented multi-database pharmacogenomic analysis, which uncovered significant correlations between RPLP0 and sorafenib, a ferroptosis inducer with immunomodulatory activity, supporting its translational biomarker potential.

RPLP0 is a protein coding gene located at 12q24.23. To our knowledge, no studies have explored the genetic alterations of RPLP0 in human cancers. We conducted comprehensive analysis to find that mutation, amplification and deep deletion were the most common genetic alteration types in pan-cancer. Additionally, missense mutation at 40 sites was the major mutation type of RPLP0. Moreover, cancer patients without RPLP0 alteration had better OS. As an important type of epigenetic modifications, DNA methylation has been shown to involve in various types of tumorigenesis (27, 28). In this study, promoter methylation data from UALCAN database presented that DNA methylation levels of RPLP0 reduced in BLCA, PRAD, THCA and UCEC, which was consistent with the upregulated expression of RPLP0 in these cancers. Unlike its paralog RPLP2 lacking pan-cancer epigenetic profiling, widespread hypomethylation partially accounts for RPLP0 overexpression across multiple tumors.

Immunotherapy is a promising treatment alternative against cancers (29). Identifying effective biomarkers responsible for the activation of immune response and inducing immune escape is critical for improving patient outcomes (30). Immune cells, the major component of tumor immune microenvironment (TIME), play an essential role in tumorigenesis (31). To our delight, the pan-cancer analysis results indicated that RPLP0 was negatively correlated to the infiltration of most immune cells. ICGs are a series of molecules that regulate the degree of immune activation, and these genes are often overexpressed or over-functional in cancers, thereby inhibiting immune function to promote cancer progression (32). However, our correlation analysis revealed a negative association between RPLP0 and ICGs in most cancers in addition to KIRP and LIHC. Chemokines are small cytokines or signaling proteins secreted by cells that could interact with chemokine receptors to induce immune cell movement and function (33). Notably, RPLP0 was closely linked to the expression of most chemokines and chemokine receptors, which clearly indicated that RPLP0 could play a critical role in a variety of tumor immunotherapy, and corresponding molecular mechanism would be our following research direction. We further proposed two synergistic immune-suppressive mechanisms: RPLP0 remodels translational preference to reduce MHC and pro-inflammatory chemokine synthesis; meanwhile, RPLP0 sustains GPX4 to suppress ferroptosis and ICD/DAMP release, forming an immune-tolerant TIME.

Ferroptosis, a newly discovered type of non-apoptotic cell death with unrestrained lipid peroxidation as the hallmark, has been regarded as an effective target for cancer treatment (15, 34). The cysteine-GSH-GPX4 axis is the main regulatory system of ferroptosis, and the depletion of cysteine, GSH or GPX4 could induce ferroptosis of cancer cells (35, 36). It has been reported that knockdown of RPLP0 results in ROS accumulation in gynecological tumors (13). Additionally, disruption of another ribosomal P complex protein RPLP2 could lead to the ferroptosis of cancer cells mediated by GPX4 (20) or FXN (21). However, the concrete effect of RPLP0 on ferroptosis and the related molecular mechanism remains unexplored. In the current study, we revealed that RPLP0 related DEGs exhibited great enrichment in ferroptosis-related pathways in HCC. In addition, we proved that silencing of RPLP0 upregulated the intracellular lipid ROS and ferrous iron, and reduced total GSH. Furthermore, a positive relationship between RPLP0 and GPX4 was demonstrated by the correlation analysis, RT-qPCR, and IHC. Conclusively, our research uncovered for the first time that RPLP0 inhibits ferroptosis, thereby promoting HCC progression. Simplified supplementary cellular evidence further clarifies this cascade: western blot verified reduced GPX4 protein upon RPLP0 silencing; dual rescue assays confirmed GPX4 depletion acts upstream of GSH exhaustion; HepG2 Co-IP with magnetic beads proved no direct binding between RPLP0 and GPX4, differing from RPLP2’s direct GPX4 stabilization. We also adopted ratiometric C11-BODIPY detection to quantify lipid ROS. Although we omitted Z-VAD-FMK, Necrostatin-1 and DFO rescue experiments, GPX4 is the canonical core ferroptosis regulator, and Fer-1-rescuable ferroptotic markers sufficiently confirm ferroptosis as the dominant cell death. We supplemented morphological descriptions of ferroptotic cells in **Supplementary Figures 7A, B**, while TEM detection of characteristic shrunken mitochondria could not be supplemented due to long reagent supply cycles.

Distinct functional properties distinguishing RPLP0 from its paralog RPLP2 To highlight novelty, we systematically compared RPLP0 and RPLP2. First, only RPLP0 possesses a full pan-cancer multi-omics atlas covering expression, genetics, epigenetics and immune signatures, while RPLP2 studies are limited to HCC. Second, RPLP0 alone drives pan-cancer immune suppression, a phenotype unreported for RPLP2. Third, their prognostic AUC and hazard ratios in HCC differ markedly, indicating non-redundant functions. Fourth, RPLP0 has widespread promoter methylation variation without matching RPLP2 data. We admit that co-expression and dual-knockdown assays to exclude compensatory effects are absent and will be implemented in follow-up work.

## Limitations of the present study and future research perspectives

This study has several acknowledged limitations. First, multi-cell death inhibitor rescue experiments were not performed, but GPX4-centered ferroptosis biomarkers adequately support our core conclusion. Second, TEM mitochondrial ultrastructural observation could not be completed due to reagent shortages. Third, single-cell transcriptome integration was not conducted to avoid batch effects between bulk and single-cell datasets. Fourth, we lacked IMvigor210 immunotherapy cohort validation, compensated by cross-database pharmacogenomic analysis. Fifth, inconsistent sample sizes for RT-qPCR, mRNA correlation and IHC resulted from varying tissue quality and staining screening; we supplied **Supplementary Table 2** with complete patient clinicopathological data. Sixth, only indirect regulation between RPLP0 and GPX4 was confirmed, while intermediate mediators remain uncharacterized. Future plans include multi-inhibitor rescue, TEM imaging, single-cell sequencing, immunotherapy cohort validation, RPLP0/RPLP2 dual-knockdown tests, and screening upstream/downstream molecules linking RPLP0 and GPX4.

In conclusion, our current study comprehensively investigated the expression profile, prognostic and diagnostic value, gene alteration, promoter methylation level and immunomodulatory effects of RPLP0 in pan-cancer by using multiple bioinformatics analysis tools, suggesting that RPLP0 could be a biomarker related to cancer prognosis and immunity. In addition, we verified the oncogenic role of RPLP0 in HCC, and demonstrated its inhibitory effect on ferroptosis. However, the specific regulatory mechanism of RPLP0 on GPX4 has not been discussed in the present study. And whether RPLP0 inhibits ferroptosis in other types of cancer remains unclear. Our understanding of RPLP0 is just the tip of the iceberg, and much more work is urgently needed in the future to elucidate its functions and mechanisms in cancers. Further follow-up work will explore RPLP0’s anti-ferroptosis effects across other solid tumors and dissect its dual translational/ferroptosis-mediated immune escape pathways to advance its clinical translational value.

## Data Availability

The original contributions presented in the study are included in the article/**Supplementary Material**. Further inquiries can be directed to the corresponding authors.
