# Supplementary material for: RPLP0 identified as a ferroptosis regulator in HCC through pan-cancer analysis
Source: Front Oncol. 2026 Aug 11;16:1891222. doi: 10.3389/fonc.2026.1891222 (PMC13503213; doi:10.3389/fonc.2026.1891222)
Supplement: Supplementary file 1 [file DataSheet1.docx]

Supplementary Materials for

**RPLP0 identified as a ferroptosis regulator in HCC through pan-cancer analysis**

**This file includes:**

Figures S1 to S7

Table S1 to S2

**Figure S1. The subcellular proteome of RPLP0 in cellular components.** (A) Subcellular locations of RPLP0 from COMPARTMENTS database. (B) Immunofluorescence staining of RPLP0 and microtubules in U-251MG cells obtained from HPA database.

**Figure S2. Survival analysis of RPLP0 in pan-cancer. (**A-C) Forest plots showed the relationships between RPLP0 and OS (A), PFS (B) or DFS (C) in different cancers.

**Figure S3. ROC curve of RPLP0 in pan-cancer.** (A) ACC. (B) BLCA. (C) BRCA. (D) CESC. (E) ESCA. (F) HNSC. (G) KICH. (H) KIRP. (I) LUAD. (J) OV. (K) PAAD. (L) PCPG. (M) SARC. (N) SKCM. (O) STAD. (P) THCA. (Q) THYM. (R) UCEC. (S) OSCC.

**Figure S4. DNA methylation features of RPLP0 in different cancers.** (A) COAD. (B) KIRC. (C) LUSC. (D) PAAD. (E) SARC.

**Figure S5. Analysis of the correlation between RPLP0 alteration and DSS, PFS and DFS in pan-cancer.** (A) DSS. (B) PFS. (C) DFS. *** p < 0.001, **** p < 0.0001.

**Figure S6. Correlation analysis between RPLP0 expression and immunoregulation-related genes in pan-cancer.** (A) The heatmap revealed the correlation between RPLP0 expression and immune-checkpoint-related gene expression in pan-cancer. (B, C) Correlation analysis between the expression of RPLP0 and chemokines (B) or chemokine receptors (C) based on the TISIDB database. ns nonsignificant (p > 0.05), * p < 0.05, ** p < 0.01.

**Figure S7. Depletion of RPLP0 enhances ferroptosis in HCC cells.** (A, B) The HepG2 (A) and Hep3B (B) cells with RPLP0 deletion treated with RSL-3 were presented by representative images. (C-G) Correlations between RPLP0 and ferroptosis-related genes including GPX4 (C), BECN1 (D), IREB2 (E), NCOA4 (F), and SLC7A11 (G) in HCC based on FerrDb.

**Table S1. The sequences of the primers for RT-qPCR used in this study.**

| Name | Forward premier (5’-3’) | Reverse premier (5’-3’) |
| --- | --- | --- |
| GPX4 | ACAAGAACGGCTGCGTGGTGAA | GCCACACACTTGTGGAGCTAGA |
| RPLP0 | TGGTCATCCAGCAGGTGTTCGA | ACAGACACTGGCAACATTGCGG |
| β-actin | CACCATTGGCAATGAGCGGTTC | AGGTCTTTGCGGATGTCCACGT |

**Table S2. The abbreviation of 36 cancer types analyzed in the study.**

| **Abbreviation** | **Cancer types** |
| --- | --- |
| ACC | Adrenocortical carcinoma |
| BLCA | Bladder Urothelial Carcinoma |
| BRCA | Breast invasive carcinoma |
| CESC | Cervical squamous cell carcinoma and endocervical adenocarcinoma |
| CHOL | Cholangiocarcinoma |
| COAD | Colon adenocarcinoma |
| COADREAD | Colorectal cancer |
| DLBC | Lymphoid Neoplasm Diffuse Large B-cell Lymphoma |
| ESCA | Esophageal carcinoma |
| GBM | Glioblastoma multiforme |
| HNSC | Head and Neck squamous cell carcinoma |
| KICH | Kidney Chromophobe |
| KIRC | Kidney renal clear cell carcinoma |
| KIRP | Kidney renal papillary cell carcinoma |
| LAML | Acute Myeloid Leukemia |
| LGG | Brain Lower Grade Glioma |
| LIHC | Liver hepatocellular carcinoma |
| LUAD | Lung adenocarcinoma |
| LUSC | Lung squamous cell carcinoma |
| MESO | Mesothelioma |
| NSCLC  OSCC | Non-small cell lung cancer  Oral Squamous Cell Carcinoma |
| OV | Ovarian serous cystadenocarcinoma |
| PAAD | Pancreatic adenocarcinoma |
| PCPG | Pheochromocytoma and Paraganglioma |
| PRAD | Prostate adenocarcinoma |
| READ | Rectum adenocarcinoma |
| SARC | Sarcoma |
| SKCM | Skin Cutaneous Melanoma |
| STAD | Stomach adenocarcinoma |
| TGCT | Testicular Germ Cell Tumors |
| THCA | Thyroid carcinoma |
| THYM | Thymoma |
| UCEC | Uterine Corpus Endometrial Carcinoma |
| UCS | Uterine Carcinosarcoma |
| UVM | Uveal Melanoma |
