# Supplementary material for: RPLP0 identified as a ferroptosis regulator in HCC through pan-cancer analysis
Source: Front Oncol. 2026 Aug 11;16:1891222. doi: 10.3389/fonc.2026.1891222 (PMC13503213; doi:10.3389/fonc.2026.1891222)
Supplement: Supplementary file 2 [file DataSheet3.docx]

Co-IP

IP-GPCX4

IB:GPX4

IB:RPLP0

IP-RPLP0

IB:GPX4

IB:RPLP0

si-RPLP0

HepG2

IB:RPLP0

IB:GPX4

IB:β-ACTIN

Hep3B

IB:RPLP0

IB:GPX4

IB:β-ACTIN
